# Global warming and warning

**DOI:** 10.6061/clinics/2019/e1219

**Published:** 2019-07-03

**Authors:** Nelson A Rosario, Gennaro D'Amato, Ignacio Ansotegui

**Affiliations:** I Departamento de Pediatria, Universidade Federal do Parana, Curitiba, PR, BR; II Division of Respiratory and Allergic Diseases, Hospital A. Cardarelli, Naples, Italy; III Allergy, Hospital Quironsalud Bizkaia, Bilbao, Spain

Evidence that the earth's temperature is increasing is provided by warming of the oceans, the melting of glaciers, rising sea levels and the diminished snow cover in the Northern Hemisphere. Climate change is associated with the intensity and frequency of precipitation, thunderstorms, sandstorms, and extreme weather events, such as heat waves, droughts, blizzards, floods, and hurricanes. Air pollution, especially increased carbon dioxide concentrations, is the driving power of the earth's warming through the greenhouse effect. Wildfires and deforestation also contribute to global warming 1.

A paper published in NEJM 2 reviewed the adverse effects of climate change on human health. Moreover, an editorial called for physicians to take a leading role in confronting climate change with the urgency that it demands 3.

Examples of how air pollution and climate change can affect allergenic plants and pollen distribution include the following: plants growing faster and an increased number of plants; increasing numbers of robust allergenic plants and an increase in aeroallergen load for patients with inhalant allergy; and an earlier and longer pollen season, as shown by phenology observations. The consequences of climate change for patients with seasonal allergic rhinoconjunctivitis and asthma are more intense symptoms and the need for more medication. Insect allergy may be more frequent and severe due to the introduction of new species or the migration of stinging and biting insects into new environments. New food proteins might also give rise to food allergies 4,5.

A global monitoring system dedicated to tracking the health dimensions of pollution and the effects of climate change on health discussed the five following domains: climate change impacts, adaptation, mitigation actions, economics, and public and political engagement 6.

The World Allergy Organization (WAO) as an institution is active through a committee on climate change promoting worldwide education of the effect of global warming on respiratory health. In 2018, a joint congress of the WAO and the American Academy of Allergy Asthma and immunology (AAAAI) focused on global environmental change and respiratory health. Both the WAO and the AAAAI could be considered resources for physicians' responses to climate change.

We agree that individual and institutional actions should be taken to reduce the substantial increases in morbidity and mortality due to anthropogenic disasters. Physicians should take the lead to promote actions to mitigate air pollution and the global warming consequences of the greenhouse effect.
